# Energy efficiency in ROS communication: a comparison across programming languages and workloads

**DOI:** 10.3389/frobt.2025.1548250

**Published:** 2025-04-02

**Authors:** Michel Albonico, Manuela Bechara Cannizza, Andreas Wortmann

**Affiliations:** ^1^ IntelAgir Research Group, Informatics Department, Federal University of Technology, Paraná (UTFPR), Francisco Beltrão, Brazil; ^2^ Institute for Control Engineering of Machine Tools and Manufacturing Units (ISW), University of Stuttgart, Stuttgart, Germany

**Keywords:** ROS, energy efficiency, programming language, ROS communication, robotic

## Abstract

**Introduction:**

The Robot Operating System (ROS) is a widely used framework for robotic software development, providing robust client libraries for both C++ and Python. These languages, with their differing levels of abstraction, exhibit distinct resource usage patterns, including power and energy consumption–an increasingly critical quality metric in robotics.

**Methods:**

In this study, we evaluate the energy efficiency of ROS two nodes implemented in C++ and Python, focusing on the primary ROS communication paradigms: topics, services, and actions. Through a series of empirical experiments, with programming language, message interval, and number of clients as independent variables, we analyze the impact on energy efficiency across implementations of the three paradigms.

**Results:**

Our data analysis demonstrates that Python consistently demands more computational resources, leading to higher power consumption compared to C++. Furthermore, we find that message frequency is a highly influential factor, while the number of clients has a more variable and less significant effect on resource usage, despite revealing unexpected architectural behaviors of underlying programming and communication layers.

## 1 Introduction

Robots play an important role in many areas of our society. They are commonly used in manufacturing, medicine, transportation (including self-driving vehicles), and as domestic allies (e.g., vacuum cleaners) (Ciccozzi et al., 2017). A great part of those robots depends on increasingly complex software, for which the Robot Operating System (ROS) (Stanford Artificial Intelligence Laboratory, 2024; Steve, 2011) is one of the most important frameworks.

ROS is considered the *de facto* standard for robotic systems in both, research and industry (Koubaa, 2015). It provides an abstraction layer that enables specialists from different areas to integrate their software into one robotic system. In addition, ROS comprises a comprehensive set of open-source libraries and packages. With over half a billion ROS packages downloaded in 2020, it has also significantly encouraged code reuse (Stanford Artificial Intelligence Laboratory et al., 2024). ROS currently has two main versions, ROS one and ROS 2, with end-of-life of ROS 1 being set to 2025. In this paper, we focus only on ROS 2, the only supported distribution in the near future, using ROS as nomenclature.

Software energy efficiency has been a recurrent concern among software developers (Pinto and Castor, 2017). This is stimulated by factors that include environmental impact, budget, and battery-dependent devices (Steve, 2011; Swanborn and Malavolta, 2020), which also applies to the robotic domain. Simple software architectural decisions can make an impact on the energy efficiency of robotic software (Chinnappan et al., 2021), where the programming language is known to be a determinant factor (Pereira et al., 2017; Albonico et al., 2024). In the case of ROS, C++, and Python are the two main programming languages thoroughly supported and documented by the community. Therefore, practitioners tend to start by choosing one of them, which currently must be done with a limited understanding of their impact on ROS systems’ energy efficiency.

In **this paper**, we conduct a systematic analysis of the energy consumption associated with message exchanges among ROS nodes implemented in C++ and Python. This study builds upon our previous work (Albonico et al., 2024), which presented initial findings on the energy impact of implementing ROS nodes in different programming languages, motivating further investigation. In that study, we observed two key challenges: (i) Python nodes exhibited higher resource usage, resulting in reduced energy efficiency, and (ii) high message frequencies constrained scalability across multiple nodes. However, the experiments were limited in scope, with only a few independent variables, which were randomly defined. To address these limitations, this paper extends the investigation by exploring four independent variables: (i) the programming language of the ROS nodes; (ii) the ROS communication pattern^1^ (e.g., topic, service, or action); (iii) the frequency of message exchange; and (iv) the number of clients/subscribers per server/publisher. Each algorithm in the study is adapted from concrete examples on the ROS tutorials Wiki page^2^, carefully adapted for this study. The experimental **results** revealed the programming language and message frequency as consistent key factors influencing energy efficiency across different ROS communication patterns. Additionally, the number of clients had an impact on power consumption, particularly for server/publisher nodes, although to a lesser degree. Interestingly, increasing the number of clients/subscribers sometimes resulted in unexpected behaviors, such as reduced power consumption in client nodes. This observation raises important questions that foster further investigation.

The **target audience** for this study includes researchers and practitioners involved in developing ROS-based systems. This work provides valuable insights to help optimize ROS systems, make informed design decisions, and conduct experiments in energy-efficient robotic systems. It encourages researchers to focus their further studies, which may consider other ROS architectural models, such as multi-node composition within single processes. Additionally, it supports practitioners in selecting suitable programming languages for their specific robotics projects, thereby contributing to the development of greener robotic software.

This paper **contributes** with insights into the energy consumption and power of ROS nodes communication across different paradigms and programming languages. It can used as a source of inspiration for developing greener robotic software, promoting environmentally conscious practices in robotic software development. It also provides a methodological framework and practical guidance for conducting further experiments in this field. Additionally, we provide a complete replication package and experimental data to benefit both researchers and practitioners. Finally, despite the relevant energy-related results, some combination of independent variables resulted in unexpected behaviors that must be shared with ROS community and can lead to important improvements ROS 2 software layers.

## 2 Background

This section presents the fundamental concepts of ROS, its communication and programming premises, and discusses Running Average Power Limit (RAPL)^3^ for energy consumption measurements.

### 2.1 Robot operating system (ROS)

ROS is a standard robotics framework in both, industry and research, for the effective development and building of a robot system (Santos et al., 2016). Currently, there are many distributions of ROS available, grouped into two main versions (ROS 1 and ROS 2). ROS 2 completely changed the architecture compared to the first version, which now massively relies on the decentralized Data Distribution Service (DDS) (Pardo-Castellote, 2003).

A ROS software architecture consists of four main types: *nodes*, *topics*, *actions*, and *services*. *Nodes* are executable processes, usually implementing a well-defined functionality of a ROS system, which can communicate asynchronously or synchronously. Asynchronous communication relies on the *publisher*/*subscriber* pattern, while asynchronous communication can be implemented over *services* or *actions*. All three communication patterns are presented in the sequence.

**FIGURE 1:**
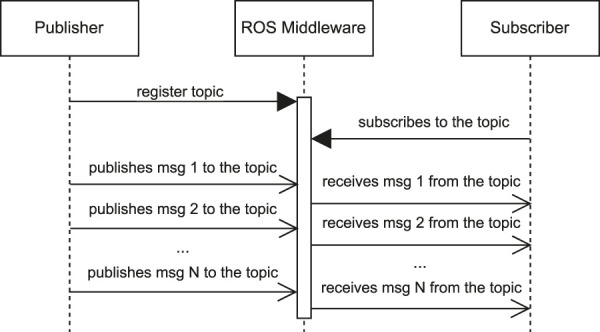
UML sequence diagram for *publisher*/*subscriber* communication.

Figure 1 depicts a Unified Modeling Language (UML) sequence diagram that represents *publisher*/*subscriber*-based ROS communication. In this communication model, the *Publisher* sends messages to a *topic*, and the *ROS Middleware* routes these messages to subscribed nodes (represented by the *Subscriber* component). This is a unidirectional flow commonly used for continuous data streams, such as sensor data^4^.

**FIGURE 2:**
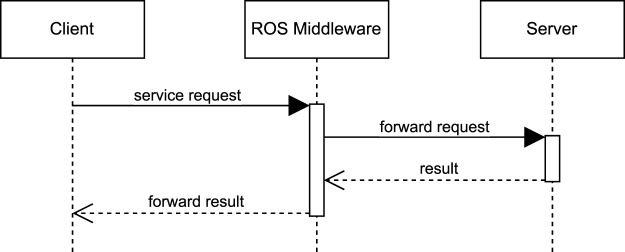
UML sequence diagram for *service* communication.

Figure 2 depicts a Unified Modeling Language (UML) sequence diagram that represents *service*-based ROS communication. The diagram captures the synchronous nature of services, where a *client* sends a one-time request to the *server* via the *ROS Middleware*. The *server* processes the request and sends the result back to the *client*. This direct kind of interaction makes services suitable for operations that trigger specific robotic actions, such as manipulating an object with a gripper, which requires a synchronous response to identify whether the operation was successful or not.

**FIGURE 3:**
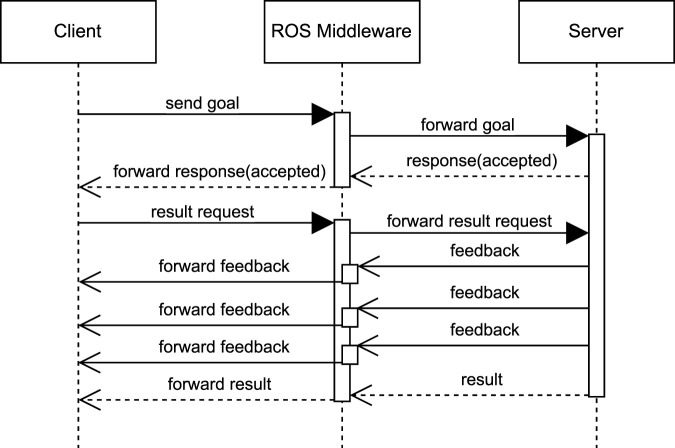
UML sequence diagram for *action* communication.

Figure 3 depicts a Unified Modeling Language (UML) sequence diagram that represents *action*-based communication in ROS. The diagram features two primary components: the *action client* and the *action server*. The *client* sends a *goal* task to the *server*, which optionally accepts it. Once the goal is accepted, the *client* requests the *result* of the task. While the task is in progress, the *server* can send periodic *feedback* to the *client*, providing updates on the task’s status. When the task is completed, the *server* sends the final *result* to the *client*. This interaction can be applied in navigation scenarios, where a navigation goal is sent to the robot. During the navigation process, the robot provides status updates, and once the task concludes, it notifies whether the goal was reached or the task failed.

#### 2.1.1 ROS programming

**FIGURE 4:**
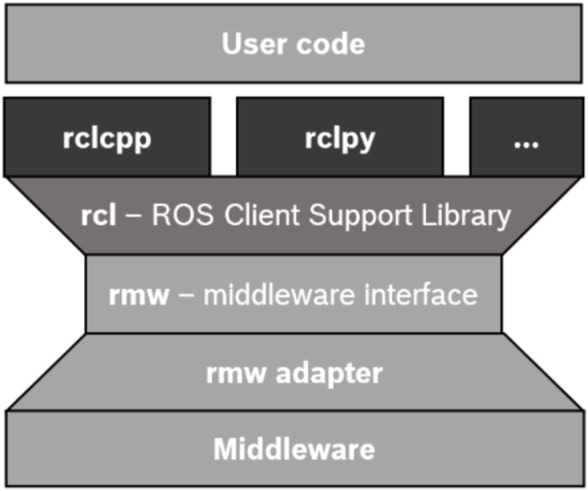
Underlying layers of a ROS node programming^7^.

ROS is recognized for its flexibility in supporting multiple programming languages, allowing developers to choose the language that best suits their needs. As depicted in Figure 4, all client libraries in ROS share the same underlying software layers. From a bottom-up perspective, this architecture begins with the communication *middleware* and *rmw adapter* (ROS Middleware Adapter), which together enable the use of various middleware solutions without requiring modifications to ROS 2 itself. Above the *rmw adapter*, the *rmw* layer serves as an interface between the lower and upper layers. At the top of this stack, the rcl layer provides a high-level API for programming ROS applications. Finally, language-oriented libraries, such as *rclcpp* ^5^ and *rclpy* ^6^, lie over the *rcl* layer, enabling developers to create ROS 2 algorithms in their chosen language.

### 2.2 Running average power limit

Modern processors provide a Running Average Power Limit (RAPL) interface for power management, which reports the processor’s accumulated energy consumption, and allows the operating system to dynamically keep the processor within its limits of thermal design power (TPD)^8^. RAPL is a recurrent profiling tool in previous related work (Zhang and Hoffman, 2015; Hähnel et al., 2012; Khan et al., 2018; von Kistowski et al., 2016). It keeps counters that can provide power consumption data for both, processor and primary memory. CPU is proven to be one of the most energy-consuming parts of a computer system (Hirao et al., 2005; von Kistowski et al., 2016; Pereira et al., 2017). Despite the primary memory usage not being a usual determinant factor in other studies (von Kistowski et al., 2016; Pereira et al., 2017), it is one of the main RAPL metrics and in this work will be used to determine whether it is still the case for ROS programming.

There are different RAPL-based energy profilers publicly available, among which PowerJoular (Noureddine, 2022) stands out. It has been recurrent in energy-efficiency studies in the literature (Thangadurai et al., 2024; Noureddine, 2024; Yuan et al., 2024). PowerJoular offers real-time insights into energy consumption patterns across diverse hardware components, such as CPUs, GPUs, and memory subsystems. Additionally, it facilitates granular energy measurements of running processes, enabling precise analysis of the energy consumption of individual ROS 2 components.

## 3 Experiment definition

The experiment of this paper is defined after the Goal Question Metric (GQM) model (Basili et al., 1994). It starts with a well-defined *goal*, which is then refined into *research questions* that are answered by measuring the software system using objective and/or subjective *metrics*.

### 3.1 Study goal

This study ***goal*** is to ***analyze*** *ROS programming with C++ and python languages* ***for the purpose*** *of understanding the extent* ***with respect to*** *energy efficiency* ***from the point of view*** *robotics researchers and practitioners* ***in the context of ROS*** *nodes communication patterns*.

### 3.2 Questions

From our goal, we derive the following research questions.

• RQ1: How is the energy efficiency of each ROS communication pattern?

In ROS, the asynchronous pattern implemented through *topics* is a common and straightforward method for message exchange among nodes. However, the other two synchronous patterns, *service* and *action*, provide essential features enabling advanced synchronization and reliability. Since synchronous communication patterns rely on session-oriented connections, they are expected to consume more computational resources. However, the impact of these design choices on the energy efficiency of ROS systems remains unexplored.

• RQ2: How do the C++ and Python implementations affect resource usage and energy consumption when handling different communication patterns among ROS nodes?

The motivation for this research question is that each language depends on its canonical client library, i.e., rclcpp and rclpy. Despite those libraries being developed following the same design principles, both languages have distinct concepts, such as compiled vs. interpreted, multi-threading management, abstraction level, etc., that may lead to particular implementations, and impact resource usage and energy consumption.

• RQ3: How does language efficiency scale over different frequencies of communication and number of clients?

Since communication is largely managed by lower-level layers, such as the DDS (see Figure 4), the efficiency differences between languages in simple examples may be minimal. However, message packing and unpacking are processed locally, which can impact both, resource usage and energy efficiency. Additionally, the number of clients can trigger multi-threading, a feature worth investigating, particularly given Python’s limitations. Python native multi-threading is limited by its Global Interpreter Lock (GIL)^9^, so achieving full parallelism often requires external libraries.

### 3.3 Metrics

Table 1 describes the metrics used for measurements during the experiments. *Energy consumption*, *power* and *execution time* are the key metrics used to assess the energy efficiency of a ROS node, while *CPU* and *memory usage* are metrics that help us to understand how intensive is the ROS node in terms of computational processing, and then reason about the measured *energy efficiency*.

**TABLE 1:** Experiment metrics.

Metric	Unit	Description
Energy Consumption	Joules (J)	Amount of energy necessary to run the ROS node
Power	Watts (W)	Energy consumption rate when running the ROS node
Execution time	Milliseconds (ms)	Total time spent to run a ROS node
CPU usage	Percentage (%)	Average CPU percentage used during a ROS node execution
Memory usage	Kilobytes (KB)	Amount of memory used during a ROS node execution

All the measurements refer to the ROS node operating system process. The energy consumption measurements take into account the two main processing factors: CPU and memory. After the energy consumption is measured, we calculate the *power* with the following formula: $P=\frac{EC}{t}$ , where P is power, EC is energy consumption, and t is the total ROS node execution time in seconds. Power measurements help identify transient effects that energy consumption (a cumulative metric) might mask. We give more details of the measurement process and tools in Section 5.3.

## 4 Experiment planning

The experiment depends on six algorithms that cover the three ROS nodes’ communication patterns (i.e., *topics*, *services*, and *actions*), implemented in both languages, Python and C++. The algorithms are based on ROS Tutorials Wiki pages^10^, which provide concise examples. They are all independent from a physical robot, seeking full controllability during the experiments.

### 4.1 ROS 2 algorithms

Table 2 depicts the six algorithms, with a short description, details of their implementation, their dependencies, and their complexities (i.e., logical lines of code–LLOC, and the algorithm McCabe’s cyclomatic complexity–MCC), the last two, for a matter of illustration of the compatibility between Python and C++ algorithm implementations. For the implementation, we began with the Python version of each algorithm, as it is the language we are most familiar with. Subsequently, we used the ChatGPT tool^11^ (GPT-4o version) to generate compatible C++ versions, which we manually reviewed to ensure compatibility and correctness.

**TABLE 2:** ROS2 algorithms subject of investigation with their dependencies and complexity measurements.

Node	Description	Main dependencies	LLOC python	LLOC C++	MCC python	MCC C++
*1.Publisher*	ROS node that continuously sends messages to a *topic*	rclpy/rclcpp, std_msgs	45	58	2.3	1.7
*2.Subscriber*	ROS node that subscribes to the *topic* and reads the published messages	rclpy/rclcpp, std_msgs	52	67	2.2	1.8
*3.Service Server*	ROS node that provides a service	rclpy/rclcpp, example_interfaces	40	67	1.8	1.8
*4.Service Client*	ROS node that consumes the *server* service	rclpy/rclcpp, example_interfaces	52	82	2.2	3.5
*5.Action Server*	ROS node that receives a goal and returns its lifetime state feedback	rclpy/rclcpp, action_tutorials_interfaces	53	85	2	1.3
*6.Action Client*	ROS node that sends the goal to the *server*	rclpy/rclcpp, action_tutorials_interfaces	36	96	1.2	3.2

It is evident that the C++ implementations resulted in greater LLOC, particularly for the *action server* and *action client*, where the difference compared to Python nearly doubled, as highlighted in red. It is important to note that exact equality is not possible due to the inherent differences between the languages. Despite variations in code size, all the algorithms exhibit similar complexity (and exactly the same for *service server*, as highlighted in blue), reflecting their overall similarity. The larger difference observed in the size and complexity of *action client* implementation is due to that node being folded into two *services* (one for sending the task and another for retrieving the result) as well as *topic* communication (for receiving task feedback). The size difference is compatible with the other algorithms if we consider the sum of the difference between the *service client* and *subscriber*, for example,. The complexity is similar to the *service client*, and could not be reduced due to the complexity of synchronizing the actions’ execution callbacks in C++. Furthermore, all the algorithms lie in the complexity range 1–10 which classifies them as simple (Thomas, 2008).

The table presents the algorithms in pairs, as they execute in the experiments (see Section 5.2). Algorithms 1 and 2 implement the *publisher* and *subscriber* pair, which enable the *publisher* to exchange different message types with the *subscriber* over a specific *topic*. Algorithms 3 and 4 implement the *service server* and *service client* pair, where the *service client* requests the *service server* to do a simple calculation of adding two integer numbers and receives its response. Algorithms 5 and 6 implement the *action server* and *action client* pair, where the *action client* sends a *task* to the *action server* (i.e., to calculate a Fibonacci sequence) via a *service goal*, receives each value of the sequence via a *feedback topic*, and at the end, receives the notification of the task completeness via a *result service*.

### 4.2 Experiment variables

Table 3 summarizes the variables used in the experiments. It categorizes the variables into three main groups: *independent*, *static*, and *dependent* variables.

1. *Independent variables*: these are the variables that we control during the experiment. They include the *ROS algorithm pair*, which refers to the specific pairs of algorithms that implement different communication patterns in ROS; the *message interval*, which defines the time gap between message exchanges; the *number of clients*, which specifies how many subscribers or clients are interacting with the server or publisher; and the *programming language* used to implement the ROS algorithms.
2. *Static variables*: these are the variables that do not change during the experiments. They include the *ROS distribution* in the Docker containers (where the ROS algorithms run), and the *environment setup*, which refers to the computer and Docker setup for the experiments.
3. *Dependent variables*: these are the measurements during experiment execution, which use the metrics previously described in Table 1. They include *CPU usage*, *memory usage*, and *energy consumption.* They reflect the system’s performance in terms of resource utilization, providing insight into how different algorithm pairs and configurations affect the overall efficiency of the system.

**TABLE 3:** Experiment variables.

Type	Name	Category	Description
*Independent variables*	ROS Algorithm Pair	Nominal	The pairs of algorithms subject to this study, which implement the different ROS communication patterns
	Message Interval	Ratio	The interval between each message exchange
	Number of Clients	Ratio	The number of *clients*/*subscribers* for each *server*/*publisher*
	Programming Language	Nominal	The programming language used to implement the ROS algorithm pairs
*Static variables*	ROS distribution	Nominal	ROS distribution on the Docker containers used for running the experiments
	Environment Setup	Nominal	The computer machine and Docker environment where the experiments are run
*Dependent variables*	CPU usage	Ratio	Average percentage of CPU usage during a experiment run
	Memory usage	Ratio	Average percentage of memory usage during a experiment run
	Energy consumption	Ordinal	Average energy consumption during a experiment run

Table 4 presents the values of the experimental variables. The *pairs of algorithms* and the *programming languages* have been discussed previously. The *message interval* ranges from 0.05 (20 messages per second) to 1.0 (1 message per second). These intervals have been selected with real-world applications in mind, where critical robotic tasks such as navigation and telemetry typically require short intervals (e.g., the *joystick* package by default relies on 20 messages per second^12^). In contrast, less time-sensitive applications, such as monitoring systems, can tolerate moderate rates (0.5–1.0 s). Longer intervals, which might be suitable for logging applications, are not considered as extended message intervals tend to lead to inexpressive resource usage. The *number of clients* increases gradually from 1 to 3, a range that is realistic for small to medium-sized robotic applications on GitHub^13^. This range also allows us to get insights into how the algorithm’s efficiency scales.

**TABLE 4:** Independent variable values.

Variable name	Values
ROS Algorithm Pair	[*publisher*, *subscriber*], [*service server*, *service client*], [*action server*, *action client*]
Message Interval (s)	0.05, 0.1, 0.2, 0.5, 1.0
Number of Clients	1, 2, 3
Programming Language	*Python*, *C++*

The factors of this study are the four independent variables, with two–three values each, where the number of treatments can be calculated as following:

$$\text{Number of Treatments}=\overset{k}{\underset{i=1}{\prod }}L_{i}=L_{1}\times L_{2}\times \cdots \times L_{k}$$

where:

$$\begin{array}{ccc} L_{1} & =3\left(\text{ROS Algorithm Pair}\right),\;L_{2} & =5\left(\text{Message Interval}\right)\\ L_{3} & =3\left(\text{Number of Clients}\right),\;L_{4} & =2\left(\text{Programming Language}\right) \end{array}$$

Thus, the total number of treatments is:

3×5×3×2=90

The treatments are repeated multiple times (see Section 5.2) to enable statistical inference from the measurements. We provide additional details regarding the experiment execution in the following section.

## 5 Experiment execution

In this section, we define hardware and software components used in the experiments and detail how the algorithms are orchestrated. For a matter of transparency and reuse, we also provide a public replication package^14^.

### 5.1 Instrumentation

**FIGURE 5:**
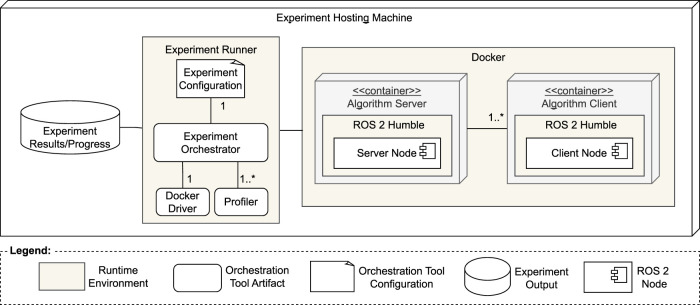
Adapted UML deployment diagram of experiment instrumentation.

Figure 5 illustrates the deployment of the experimental artifacts on a single desktop computer with the following specifications: Linux Ubuntu 22.04 operating system, kernel version 6.2.0–33-generic, 20 GB of RAM, and an Intel(R) Core(TM) i5-10210U CPU at 1.60 GHz. Each algorithm was implemented as a ROS 2 node using the ROS Humble distribution^15^, which has an end-of-life (EOL) date in May 2027, and distributed as part of a single ROS 2 package with all the implementations. In the experiments, each ROS node runs in a separate Docker (version 24.0.7) container. All the procedures are inside the node’s callback functions, so ROS can spin them, taking care of underlying threading^16^. Algorithm executions are orchestrated by the ros2 run command, which speeds up automation and guarantees the same underlying layers for every execution.

To eliminate concurrency, all experiments were conducted on a dedicated machine, ensuring no other end-user applications were running. The operating system’s power-saving mode was set to performance, ensuring unrestricted power usage. This configuration was crucial to maintain a controlled environment, providing consistent priority for each execution. Additionally, we assigned a priority level of 0 (the highest as non-root) to the processes corresponding to the algorithms under experimentation, granting them priority access to the machine’s resources. Between each experiment, a 30-s interval was observed to allow the machine to cool down, which by experimental observation is enough waiting time for the CPU to return to its baseline usage percentage.

### 5.2 Algorithms execution

The algorithms are implemented in pairs, as shown in Table 2, consisting of a *publisher/server* and a *subscriber/client*. Each pair executes repeatedly according to the defined *message interval* until reaching a total run-time of 3 min. The total run-time has been carefully chosen so the ROS nodes have time to capture transient effects like initialization overhead, start-up energy spikes, and system state changes, and to average out possible transitional background processes that may insert noise to the measurements. It also makes the experiment repetitions be completed in a couple of days and enables enough data points for statistical analysis. When multiple *subscriber/clients* are present, each performs the same communication with the *publisher/server* in parallel. Each round typically takes ≈4.5 minutes on average to complete, where a complete round of the 90 treatments takes ≈405 minutes (or 6.75 h). To ensure statistical significance, each treatment is repeated 20 times, resulting in an overall execution time of ≈135 hours ( ≈5.6 days).

• For nodes 1 and 2 (cf. Table 2), the *publisher* continuously sends a preset message to the *subscriber* at the specified interval. To avoid messages to be lost in high frequency, the *subscriber* is set with a message querying of 10.
• For nodes 3 and 4, the *service client* establishes a connection with the *service server*, uses its service (e.g., performing a calculation with two integers), and receives the result. The connection remains active throughout the experiment to focus on evaluating communication exchanges.
• For nodes 5 and 6, the *action client* connects to the *action server* once at the start of the experiment. It continuously sends goals (e.g., calculating a Fibonacci sequence), receives intermediary feedback, and obtains the final sequence as the result.

### 5.3 Resource profiling

We profile energy consumption using *PowerJoular* (Noureddine, 2022), which leverages Intel’s Running Average Power Limit Energy Reporting (RAPL)^17^, measuring both, CPU utilization and energy consumption. PowerJournal is an energy monitoring tool that leverages the RAPL interface available in Intel processors to measure power consumption. RAPL provides energy estimations at different levels, such as the package (CPU socket) and the DRAM. These estimations are derived from internal processor models rather than direct physical measurements but have been shown to be accurate for comparative energy consumption analysis. PowerJournal interacts with RAPL via the powercap framework in Linux, periodically reading energy counters exposed through*/sys/class/powercap*. This allows us to measure energy consumption at fine-grained intervals with minimal overhead. For the experiments, *PowerJoular* is configured to monitor the energy usage of each ROS node’s processes individually, capturing data at a fixed rate of one measurement per second (with no option to increase the frequency). To gather more granular data on memory usage and CPU utilization, we developed a customized Python script using the *psutil* ^18^ library. This script records measurements at a rate of 10 samples per second.

### 5.4 Data analysis

We begin the data analysis by visually exploring the distribution of power consumption across different combinations of experimental factors: the programming languages Python and C++, message exchange frequency, and the number of clients. After examining the visual data representation, we proceed with a rigorous statistical testing strategy to assess and validate the primary interpretations. In the following section, we detail the statistical testing approach applied to our data, which can be replicated via replication package^19^.

#### 5.4.1 Statistical tests

The process of statistical testing starts with an evaluation of key assumptions necessary for parametric tests, such as the distribution of the data and the equality of variances across groups. This approach involves four phases, each dedicated to confirming these assumptions and determining the most appropriate test.

##### 5.4.1.1 Normality assessment

The first step is to verify whether the data follows a normal distribution, as many parametric tests, including ANOVA (St and Svante, 1989), rely on this assumption. To assess normality, we use the Shapiro-Wilk test (Shapiro and Wilk, 1965), which is particularly effective for small sample sizes. If the data does not meet normality, we apply Box-Cox transformations (George and Cox, 1964) to adjust it. Once normality is nearly achieved, we proceed with detecting and removing outliers using the Interquartile Range (IQR) method. We performed a *post hoc* analysis to assess the impact of outlier removal, and observed that this step removes only extreme values (less than 5%), where a representative part of the core dataset is still available for statistical tests.

##### 5.4.1.2 Homogeneity of variance evaluation

Next, we examine the assumption of equal variances across groups, another important condition for tests like ANOVA. Ensuring that the variances within groups are similar allows for valid comparisons. Levene’s test is applied here, as it is still consistent even with violations of normality.

##### 5.4.1.3 Parametric and non-parametric testing

When both normality and homogeneity of variance are satisfied, we proceed with the one-way ANOVA to test for differences in means across groups. If the analysis involves just two groups, the t-test is applied instead. With the violation of any of the assumptions, we rely on non-parametric alternatives, such as Welch’s ANOVA (Bernard, 1951) and the Kruskal–Wallis test (Kruskal and Wallis, 1952), which do not require normality or equal variances.

##### 5.4.1.4 Post-hoc analysis

If statistical test results suggest significant group differences, *post hoc* analysis is conducted to pinpoint where the differences occur. For parametric tests, we rely on Tukey’s Honestly Significant Difference (HSD) test (Abdi and Williams, 2010), as it accounts for multiple comparisons, reducing the risk of false positives. In cases where non-parametric tests were used, we rely on Dunn’s test (Olive Jean Dunn, 1961), which offers robustness in the face of normality violations.

## 6 Results

In this section, we present the key results of the three studied communication patterns and provide a concise discussion of the observed data based on statistical tests. Finally, we compare the measurements across the different communication patterns. All the results presented in this section, have been carefully and manually inspected, and the runs that result in unexpected measurements have all been confirmed by re-execution.

### 6.1 *Publisher* and *subscriber*

We begin the analysis with the *publisher* and *subscriber* data, first describing the mean/total values of each measurement across different configurations. Next, we illustrate the primary data distributions, followed by the presentation of the statistical testing results.

#### 6.1.1 Publisher

Table 5 summarizes the measurements of the *publisher* node across the experiments. Across all metrics, C++ demonstrates consistently superior efficiency than Python, particularly in power/energy consumption and CPU utilization (both being directly related). Python exhibits higher resource overhead, especially at high message frequencies of 0.05 and 0.1 s. Memory usage for both remains stable over different configurations, with Python resulting at approximately 41,000 KB on average, nearly double that of C++, which averages around 21,000 KB. The little memory variation across different configurations for both languages is comprehensible since the algorithms remain the same and there is only message replication, with no special pre/post-processing. Increasing the number of clients seems to raise resource consumption for both implementations slightly, and the effect appears to be less significant compared to variations caused by message interval. Furthermore, at the highest frequency (0.05-s message interval), the number of clients does not result in a consistent increasing in power consumption for both languages, despite the grow in CPU usage. However, it is not possible to observer an important decrease either. We carefully investigated the execution logs, and we could not identify any issues. Therefore, we assume this is due to the overhead of such a high-frequency message exchange.

**TABLE 5:** Results of *publisher* with different frequencies and number of clients over 20 executions.

Language	# Clients	Msg. Interval (s)	Avg. CPU utilization (%)	Avg. Memory utilization (KB)	Total CPU energy (J)	CPU power (W)
*C++*	1	0.05	0.59	21,009	48.82	0.27
		0.1	0.32	20,837	25.45	0.14
		0.25	0.13	20,908	11.1	0.06
		0.5	0.07	20,862	6.19	0.03
		1.0	0.05	20,856	4.05	0.02
	2	0.05	0.63	21,067	52.03	0.29
		0.1	0.33	21,041	26.48	0.15
		0.2	0.15	21,023	12.33	0.07
		0.5	0.08	20,991	6.95	0.04
		1.0	0.06	21,003	4.52	0.03
	3	0.05	0.68	21,086	54.53	0.3
		0.1	0.37	20,018	29.11	0.16
		0.2	0.16	21,100	13.58	0.08
		0.5	0.09	21,004	7.74	0.04
		1.0	0.06	20,985	4.96	0.03
*Python*	1	0.05	2.1	41,033	127.83	0.71
		0.1	1.02	41,093	61.20	0.34
		0.2	0.45	41,026	37.5	0.21
		0.5	0.24	40,993	19.64	0.11
		1.0	0.13	40,987	11.17	0.06
	2	0.05	2.25	41,174	118.80	0.66
		0.1	1.15	41,139	70.21	0.39
		0.2	0.49	41,090	39.09	0.22
		0.5	0.26	41,080	21.19	0.12
		1.0	0.15	41,083	11.79	0.07
	3	0.05	2.31	41,278	122.42	0.68
		0.1	1.28	37,131	79.23	0.44
		0.2	0.53	41,139	41.5	0.23
		0.5	0.28	41,158	22.64	0.13
		1.0	0.16	41,164	12.66	0.07

**FIGURE 6:**
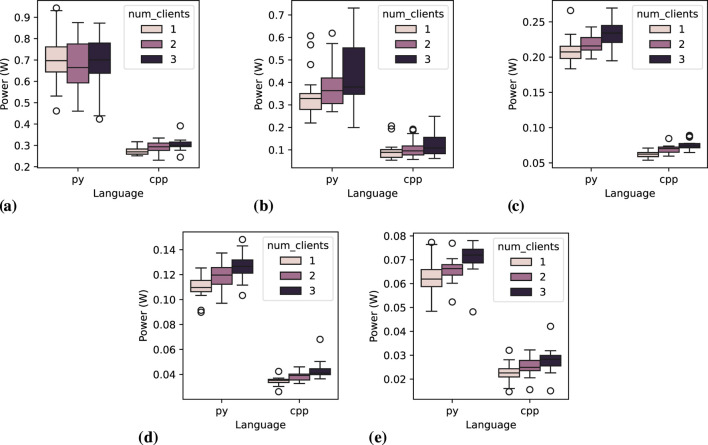
Power distribution for the *publisher* node across 20 executions, varying the number of clients and message interval. **(A)** Message interval: 0.05 s, **(B)** Message interval: 0.1 s, **(C)** Message interval: 0.2 s, **(D)** Message interval: 0.5 s, **(E)** Message interval: 1.0 s.

Figure 6 illustrates the distribution of average power consumption for the *publisher* across all repetitions. All figures show C++ with consistently lower power consumption compared to Python. It is also visually evident that shorter message intervals are associated with higher power consumption. Additionally, in most cases, power consumption tends to increase slightly with the number of clients. An exception to this trend is observed at 0.05-s message intervals (Figure 6A, where Python exhibits an anomalous behavior previously highlighted in Table 5, with a slight reduction of power consumption with two clients, and then an increase with three clients (which mean value is close to the one with one client).

##### 6.1.1.1 Statistical tests

**FIGURE 7:**
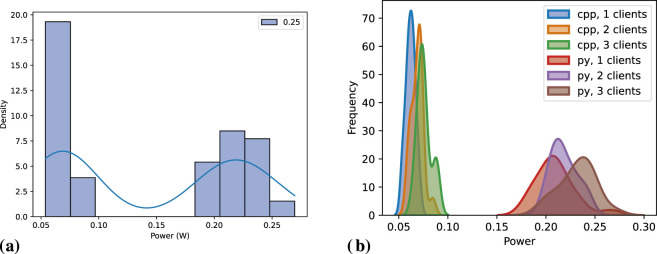
Power distribution for the *publisher* node across 20 executions, varying the number of clients at 0.2-s message interval. **(A)** Overall distribution at 0.2 s interval, **(B)** Distribution at 0.2 s interval by number of clients.

Shapiro-Wilk tests reveal a significant deviation from normality in the data when grouped by a single independent variable. For instance, in the case of the variable language, the test statistic of 0.7147 with a p-value of $3.576\times 10^{-11}$ falls far below common significance thresholds (e.g., 0.05), strongly rejecting the null hypothesis that the data follows a normal distribution. This pattern is consistent across other independent variables. This outcome aligns with the boxplots in Figure 6, which illustrate a substantial difference between Python and C++ languages. For instance, as shown in Figure 7A, when the data is grouped by a specific message interval of 0.2 s, two distinct clusters of measurements emerge. Figure 7B further reveals that these clusters correspond to groups of measurements based on different programming languages and the number of clients. This is a pattern among groups of other independent variables (what can be inspected in the replication package), where the clusters correspond to the programming languages and, when isolated, appear to follow a normal distribution. These observations strongly suggest that programming language directly influences energy efficiency. This is confirmed by Kruskal–Wallis test on groups by language (non-parametric test since data is not normally distributed), which results in an high *H* value (205.24) and a p-value $\left(1.49\times 10^{-46}\right)$ very distant from the significance threshold. Therefore, for comparative analysis assuming normal distribution, it is essential to group other variables with the programming language.

Considering the programming language as a determining factor, we conduct statistical tests involving message intervals and the number of clients, filtering the data by language (i.e., statistical tests are run for each language separately). We start by testing the effect of message intervals with Kruskal–Wallis test since not every group is normally distributed. The test reveals a significant difference among the groups for both Python $\left(p=6.53\times 10^{-60}\right)$ and C++ $\left(p=1.9\times 10^{-60}\right)$ . Table 6 summarizes the results of Dunn’s *post hoc* tests for the C++ language across the different message interval groups, with measurements for all numbers of clients. A notable observation is that comparisons between message intervals consistently display increasing differences between groups as the message interval grows. This pattern is also observed for the Python language and for both languages when operating with only one client (which helps avoid bias due to the number of clients). This confirms that the power consumption of the *publisher* node tends to be heavily influenced by the message interval.

**TABLE 6:** Dunn’s *post hoc* test results for language C++ and different message intervals, with cells in gray representing no significant statistical difference.

	0.05	0.10	0.2	0.50	1.00
0.05	$1.000000\times 10^{0}$	$1.393\times 10^{-3}$	$3.484\times 10^{-13}$	$3.487\times 10^{-29}$	$2.570\times 10^{-50}$
0.10	$1.393\times 10^{-3}$	$1.000000\times 10^{0}$	$1.636\times 10^{-3}$	$2.820\times 10^{-13}$	$2.067\times 10^{-28}$
0.2	$3.484\times 10^{-13}$	$1.636\times 10^{-3}$	$1.000000\times 10^{0}$	$1.246\times 10^{-3}$	$6.858\times 10^{-13}$
0.50	$3.487\times 10^{-29}$	$2.820\times 10^{-13}$	$1.246\times 10^{-3}$	$1.000000\times 10^{0}$	$2.585\times 10^{-3}$
1.00	$2.570\times 10^{-50}$	$2.067\times 10^{-28}$	$6.858\times 10^{-13}$	$2.585\times 10^{-3}$	$1.000000\times 10^{0}$

Kruskal–Wallis tests on group by programming language and number of clients revealed no significant differences between the groups for Python (p=0.36) and C++ (p=0.13) . However, when additionally grouping the data by message intervals, most groups exhibited statistically significant differences. The exceptions were the groups corresponding to the Python and C++ languages at a 0.05-s message interval, suggesting an overhead of the *publisher* node.

#### 6.1.2 Subscriber

Table 7 presents the performance results for a single ROS-based *subscriber* node across the experiments. Similar to the findings from the *publisher* node analysis, the Python implementation demonstrates higher CPU and memory usage compared to C++, along with greater energy and power consumption. For both languages, resource usage generally decreases with increasing message frequency, although not linearly. Exceptions are also observed at frequencies of 0.05 and 0.1 s, which exhibit an unstable trend consistent with the *publisher* results. Unlike the *publisher*, increasing the number of clients does not significantly impact resource consumption, which is comprehensible since there should be no additional work to be processed as a *subscriber*. However, especially for C++, we observe a slightly increasing pattern as the number of clients increases, which may be the result of extra synchronization work. Memory usage remains stable across all scenarios and aligns closely with the measurements for the *publisher* node.

**TABLE 7:** Comparative results of one *subscriber* node with different frequencies and number of clients over 20 executions.

Language	# Clients	Msg. Interval (s)	Avg. CPU utilization (%)	Avg. Memory utilization (KB)	Total CPU energy (J)	CPU power (W)
*C++*	1	0.05	0.57	21,291	47.4	0.26
		0.1	0.31	21,202	24.89	0.14
		0.2	0.13	21,234	10.96	0.06
		0.5	0.07	21,183	6.16	0.03
		1.0	0.05	21,144	3.9	0.02
	2	0.05	0.6	21,380	49.65	0.28
		0.1	0.33	21,355	26.09	0.15
		0.2	0.14	21,349	11.87	0.07
		0.5	0.08	21,356	6.59	0.04
		1.0	0.05	21,432	4.45	0.02
	3	0.05	0.66	21,475	53.28	0.3
		0.1	0.36	21,438	28.28	0.16
		0.2	0.15	21,388	12.81	0.07
		0.5	0.09	21,401	7.25	0.04
		1.0	0.06	21,349	4.71	0.03
*Python*	1	0.05	2.79	41,063	160.21	0.89
		0.1	1.43	41,053	90.35	0.5
		0.2	0.6	41,023	50.08	0.28
		0.5	0.31	40,889	25.39	0.14
		1.0	0.17	40,975	14.51	0.08
	2	0.05	3.08	41,120	147.60	0.82
		0.1	1.53	41,107	92.66	0.5
		0.2	0.61	40,977	48.55	0.27
		0.5	0.32	41,014	26.25	0.15
		1.0	0.18	41,127	14.51	0.08
	3	0.05	3.06	41,126	154.82	0.86
		0.1	1.7	39,193	93.21	0.52
		0.2	0.64	41,134	49.97	0.28
		0.5	0.34	41,148	27.19	0.15
		1.0	0.19	41,080	14.78	0.08

**FIGURE 8:**
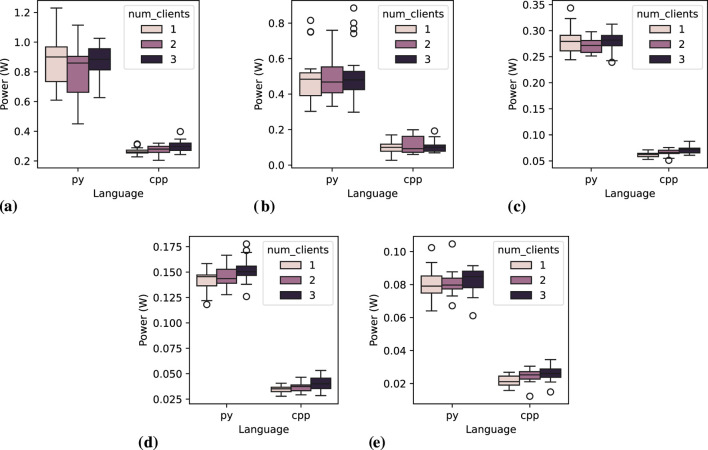
Power consumption distribution for one *subscriber* node across 20 executions, while varying the number of clients and message interval. **(A)** Message interval: 0.05 s, **(B)** Message interval: 0.1 s, **(C)** Message interval: 0.2 s, **(D)** Message interval: 0.5 s, **(E)** Message interval: 1.0 s.

Figure 8 depicts the distribution of power consumption means for a single *subscriber* across 20 executions. The instability highlighted in Table 7 is evident in Figures 8A, B. In Figures 8C, E, we observe a pattern for C++ where power consumption increases with the addition of a second client but stabilizes with a third *subscriber*. However, the difference appears minor, supporting the assumption that this is caused by overhead in the *publisher* node managing multiple subscribers. Our analysis of the code, including the *rclcpp* library, revealed no explicit synchronization mechanisms in C++ *publisher* handling multiple subscribers. It remains possible that this overhead originates from underlying layers, such as the DDS middleware. For instance, DDS might require additional internal structures and resources to manage the second *subscriber*, resulting in a one-time setup cost with no further increase when adding a third. However, further investigating this potential behavior falls outside the scope of this paper.

##### 6.1.2.1 Statistical tests

The statistical tests reveal that the data distributions closely follow the ones of the *publisher*; however, it tends to be less normally distributed which leads to some different statistical tests across groups of independent variables, as discussed in Section 5.4.1.

For programming languages, the Kruskal–Wallis test results in an *H* statistic of 205.42 and a *p-value* of $1.37\times 10^{-46}$ , indicating a significant difference between Python and C++ measurements. This finding aligns with the observations presented in Table 7 and Figure 8. A similar pattern is evident across groups of programming language and message intervals, where Kruskal–Wallis test results in an *H* statistic of 286.68 and a *p-value* of $8.09\times 10^{-61}$ for Python, and in an *H* statistic of 284.88 and a *p-value* of $1.97\times 10^{-60}$ for C++. The statistical difference is also observed when grouping programming language and different number of clients. For Python language, one-way ANOVA test results in a $p=2.52\times 10^{-8}$ , while for C++ language it results in p=0.005 .

Upon further analysis of message intervals, the one-way ANOVA reveals a statistically significant difference only for the Python language at the 0.05-s message interval $\left(p=2.62\times 10^{-8}\right)$ . However, this finding may be inconclusive, given the anomalies previously observed in the results table and Figure 8A. For the other intervals, Kruskal–Wallis tests show no significant differences among groups, with p=0.54 for the 0.1-s message interval and p=0.38 for the 1.0-s interval. Similarly, one-way ANOVA test across 0.2-s and 1.0-s message intervals indicates no statistical difference among groups, with p=0.21 for both message intervals.

Distinctly from Python, C++ *subscriber* nodes exhibit statistical differences among groups for all message intervals except the 0.1-s interval. Interestingly, the 0.1-s interval also shows the highest variation with two clients, as seen in Figure 8B. We have repeated this experiment to guarantee that this was not added by any noise, and the result is consistent among both executions. Post-hoc tests revealed that, in most cases where there is a statistical difference, it occurs between groups with one and three clients, where gradual increases in the number of clients do not result in significant statistical differences (i.e., from one to 2, and from two to three clients). The only message interval showing statistical differences across all groups is 0.2 s. Analyzing Figure 8C, this interval visually demonstrates the least variation in measurements, which likely influences the statistical outcomes.

The results and statistical tests confirm that programming language and message interval significantly impact the energy efficiency of *subscriber* nodes. In contrast, the number of clients shows only a slight impact on energy consumption, which is expected since the measurements refer to a single *subscriber* node, and the amount of messages received by that node should be independent of the number of clients.

### 6.2 Service

In this section, we present the results for *service server* and *service client* across the experiments with different independent variable combinations.

#### 6.2.1 Service server

Table 8 presents the mean values of *service server* measurements across the different combinations of independent variables. Unlike the *publisher* node in the previous results, for all the combinations, CPU usage and energy measurements increase as the number of clients grows. This behavior can be attributed to the nature of the nodes: the *publisher* node relies heavily on underlying layers, such as *rmw*, for message replication, with minimal computation in the node itself. In contrast, the *service server* node involves additional calculations, which may contribute to increased processing and, consequently, higher power consumption. Additionally, as observed in the previous communication pattern, memory usage remains stable and is approximately doubled for the Python implementation compared to the C++ implementation. At high message frequencies, the mean power consumption for C++ is less than one-third of that of Python, a difference that is also reflected in the CPU usage measurements.

**TABLE 8:** Comparative results on *service server* node with different frequencies and number of clients over 20 executions.

Language	# Clients	Msg. Interval (s)	Avg. CPU utilization (%)	Avg. Memory utilization (KB)	Total CPU energy (J)	CPU power (W)
*C++*	1	0.05	0.52	19,768	41.5	0.23
		0.1	0.29	20,696	23.11	0.13
		0.2	0.13	19,722	11.13	0.06
		0.5	0.09	20,856	7.16	0.04
		1.0	0.06	19,698	4.4	0.02
	2	0.05	0.94	19,915	70.8	0.4
		0.1	0.5	20,939	38.1	0.21
		0.2	0.22	20,825	17.52	0.1
		0.5	0.12	20,793	10.04	0.06
		1.0	0.08	20,810	6.25	0.03
	3	0.05	1.0	19,657	72.83	0.41
		0.1	0.53	20,965	40.61	0.23
		0.2	0.24	21,016	18.84	0.11
		0.5	0.14	20,959	10.95	0.06
		1.0	0.09	20,914	6.5	0.04
*Python*	1	0.05	2.43	41,041	156.92	0.88
		0.1	1.26	39,008	90.76	0.51
		0.2	0.55	41,063	40.73	0.23
		0.5	0.29	40,984	22.78	0.13
		1.0	0.17	41,053	13.41	0.08
	2	0.05	2.61	39,111	159.29	0.89
		0.1	1.39	41,049	97.23	0.54
		0.2	0.6	39,117	44.68	0.25
		0.5	0.32	41,156	25.04	0.14
		1.0	0.19	41,117	13.99	0.08
	3	0.05	2.91	39,245	171.27	0.96
		0.1	1.52	41,215	100.04	0.56
		0.2	0.66	41,137	49.11	0.27
		0.5	0.36	41,176	27.58	0.15
		1.0	0.21	41,201	15.9	0.09

Figure 8 depicts the distribution of *subscriber* power measurements. The graphs show that the programming language and message interval are key factors influencing the results. The number of clients seems to affect the two languages differently. For Python, the measurements are less predictable at high message frequencies (0.05-s and 0.1-s intervals) while we observe a clear trend for other message intervals, with power consumption increasing as the number of clients grows. In contrast, for C++, power consumption rises between one and two clients but remains relatively stable between two and three clients, suggesting a one-time effect once multiple clients are involved.

##### 6.2.1.1 Statistical tests

**FIGURE 9:**
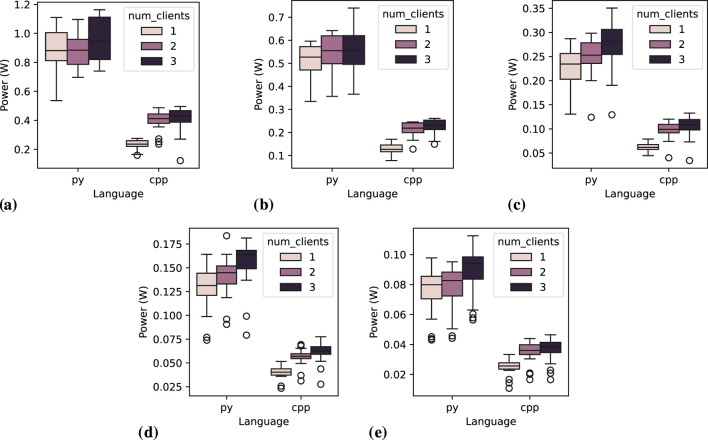
Power consumption distribution for the *service server* node across 20 executions, varying the number of clients and message interval. **(A)** Message interval: 0.05 s, **(B)** Message interval: 0.1 s, **(C)** Message interval: 0.2 s, **(D)** Message interval: 0.5 s, **(E)** Message interval: 1.0 s.

The statistical tests confirm the main findings observed in the results table and graphs. The Kruskal–Wallis test for different message intervals shows a highly significant difference for the C++ node, with $p=3.49\times 10^{-55}$ , and for the Python node, with $p=1.98\times 10^{-58}$ . These results indicate a significant difference between groups. For both languages, all pairwise comparisons in the *post hoc* Dunn’s test reveal significant statistical differences, with each group differing from the others. On the one hand, when grouping by the number of clients for Python, the one-way ANOVA fails to reject the null hypothesis (p=0.19) , suggesting no statistical difference between the groups. On the other hand, for C++, the Kruskal–Wallis test shows a statistically significant difference when grouping by the number of clients (p=2.32) . For C++ groups, however, *post hoc* Dunn’s test indicates no statistically significant difference between group 2 and group 3, only between group 1 and the others. The observation about C++ groups is also evident in Figure 9, confirming the one-time effect when adding multiple clients. That figure also supports the assumption that Python’s statistical unpredictability may be directly linked to its high variation in measurements. However, as a future work, it is important to further investigate the effect of a higher number of clients.

#### 6.2.2 Service client

Table 9 presents the mean values of *service client* measurements across various combinations of independent variables. An unexpected trend is observed for both languages, where power consumption and CPU usage decrease as the number of clients increases. This effect is more pronounced at higher message frequencies, with both measurements becoming more stable or showing no significant differences between the 0.2-s and 1.0-s message intervals. Notably, Python likely for *publisher*/*subscriber* pattern exhibits a larger variation at higher frequencies, suggesting that it tends to be less stable when handling demanding robotic communication.

**TABLE 9:** Comparative results of one *service client* node with different frequencies and number of clients over 20 executions.

Language	# Clients	Msg. Interval (s)	Avg. CPU utilization (%)	Avg. Memory utilization (KB)	Total CPU energy (J)	CPU power (W)
*C++*	1	0.05	0.47	19,724	37.68	0.21
		0.1	0.26	20,638	20.63	0.12
		0.2	0.12	19,660	9.81	0.05
		0.5	0.07	20,767	5.96	0.03
		1.0	0.05	19,685	4.85	0.02
	2	0.05	0.61	19,781	45.96	0.26
		0.1	0.33	20,933	25.26	0.14
		0.2	0.15	20,888	12.19	0.07
		0.5	0.09	20,805	7.04	0.04
		1.0	0.06	20,805	4.64	0.03
	3	0.05	0.53	20,698	39.28	0.22
		0.1	0.28	20,934	22.05	0.12
		0.2	0.14	20,882	11.21	0.06
		0.5	0.08	20,883	7.26	0.04
		1.0	0.05	20,902	5.13	0.03
*Python*	1	0.05	3.07	40,005	221.04	1.13
		0.1	1.74	39,038	124.8	0.7
		0.2	0.67	39,003	51.4	0.29
		0.5	0.37	40,033	28.9	0.16
		1.0	0.2	40,063	15.73	0.09
	2	0.05	2.86	39,397	175.16	0.98
		0.1	1.56	41,154	108.82	0.61
		0.2	0.65	39,129	55.62	0.28
		0.5	0.34	41,088	26.24	0.15
		1.0	0.18	40,168	13.9	0.08
	3	0.05	2.67	39,734	157.21	0.88
		0.1	1.32	41,424	87.01	0.49
		0.2	0.54	41,238	39.41	0.22
		0.5	0.3	41,208	22.67	0.13
		1.0	0.16	40,188	12.72	0.07

**FIGURE 10:**
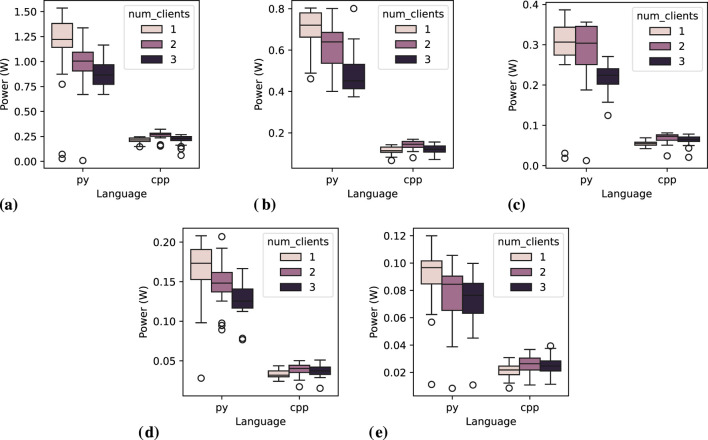
Power consumption distribution for one *service client* node across 20 executions, while varying the number of clients and message interval. **(A)** Message interval: 0.05 s, **(B)** Message interval: 0.1 s, **(C)** Message interval: 0.2 s, **(D)** Message interval: 0.5 s, **(E)** Message interval: 1.0 s.

Figure 10 shows the distribution of mean power consumption across the 20 executions for each combination of independent factors. This confirms the observation from Table 9, where Python exhibits a noticeable reduction in power consumption as the number of clients increases. In contrast, for C++ *service clients*, it is only visually evident that there is an increase in the power consumption from one to two clients, while is observed a reduction when increasing the number of clients from two to three. Additionally, Python measurements display a significant number of outlier data points, whereas C++ measurements do not show this issue. We conducted a careful investigation into the causes of the Python node’s unstable behavior but found no issues in the execution logs. We repeated the experiment without the Experiment-Runner orchestrator to avoid any possible noise, which did not change the results. Additionally, we experimented with an alternative Quality of Service (QoS) strategy, inspired by a recently reported issue on GitHub^20^. However, this adjustment did not affect the distribution of the measurements either. Based on these findings, we assume that the nodes functioned correctly and that the instability originates from a Python-related issue, which must motivate further investigation as part of future work.

##### 6.2.2.1 Statistical tests

Kruskal–Wallis test reveals a significant statistical difference between groups of message intervals for both languages ( $p=2.89\times 10^{-53}$ for Python and $p=1.44\times 10^{-57}$ for C++). Post-hoc Dunn’s test confirms that all groups are statistically different for both languages. For Python, when grouped by the number of clients, the Kruskal–Wallis test indicates a significant difference among groups (p=0.015) . However, the *post hoc* Dunn’s test shows that the statistical difference is only evident between group 1 and the others, with no significant difference between groups 2 and 3. Similarly, for C++, groups based on the number of clients also exhibit significant differences (Kruskal–Wallis test, p=0.00024 ). However, the pairwise *post hoc* test (Tukey HSD) suggests that the difference between groups 1 and 3 is not statistically significant. This observation aligns with the trends depicted in Figure 10.

### 6.3 Actions

In this section, we present the results for the *action server* and *action client*. As for the other communication pattern pairs, we provide related plots and perform statistical tests to validate our visual observations from the data representations.

#### 6.3.1 Action server

Table 10 summarizes the mean measurements of the action server node across various message frequencies and numbers of clients over 20 executions. The key observations are as follows: *CPU usage*, and consequently *CPU power*, are consistently influenced by message frequency, with notable increases at high frequencies corresponding to 0.05-s and 0.1-s intervals. Additionally, memory usage follows a pattern similar to that observed in previous server nodes. Interestingly, at a 0.05-s interval, power consumption decreases when the number of clients increases from two to three, despite CPU usage not exhibiting a corresponding decrease. Given the very low CPU power measurements, this anomaly could be attributed to external noise.

**TABLE 10:** Comparative results on *action server* node with different frequencies and number of clients over 20 executions.

Language	# Clients	Msg. Interval (s)	Avg. CPU utilization (%)	Avg. Memory utilization (KB)	Total CPU energy (J)	CPU power (W)
*C++*	1	0.05	0.32	21,680	14.85	0.08
		0.1	0.33	21,102	15.52	0.09
		0.2	0.13	21,152	7.41	0.04
		0.5	0.09	21,058	5.31	0.03
		1.0	0.07	20,084	3.79	0.02
	2	0.05	0.37	21,884	16.93	0.09
		0.1	0.36	21,590	17.16	0.1
		0.2	0.19	20,694	8.84	0.05
		0.5	0.12	20,582	5.26	0.03
		1.0	0.08	19,610	3.68	0.02
	3	0.05	0.43	21,696	19.59	0.11
		0.1	0.41	21,408	19.14	0.11
		0.2	0.31	18,826	10.03	0.06
		0.5	0.17	19,620	5.23	0.03
		1.0	0.11	18,630	3.49	0.02
*Python*	1	0.05	2.03	42,888	85.47	0.48
		0.1	1.0	40,891	45.9	0.26
		0.2	0.44	42,060	22.76	0.13
		0.5	0.25	39,720	13.96	0.08
		1.0	0.16	39,654	8.14	0.05
	2	0.05	2.15	42,872	86.08	0.48
		0.1	1.1	42,907	47.56	0.26
		0.2	0.47	42,104	23.84	0.13
		0.5	0.26	42,060	14.05	0.08
		1.0	0.16	41,865	8.43	0.05
	3	0.05	2.21	40,737	83.3	0.46
		0.1	1.13	42,915	48.09	0.27
		0.2	0.48	42,006	23.86	0.13
		0.5	0.27	42,030	13.97	0.08
		1.0	0.18	41,694	8.47	0.05

**FIGURE 11:**
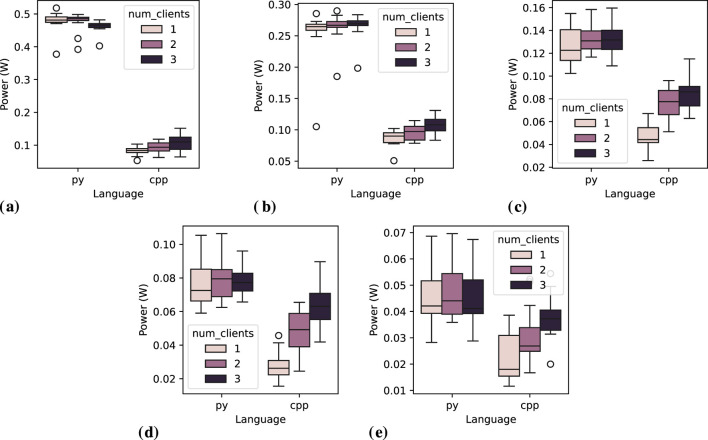
Power consumption distribution for the *action server* node across 20 executions, varying the number of clients and message interval. **(A)** Message interval: 0.05 s, **(B)** Message interval: 0.1 s, **(C)** Message interval: 0.2 s, **(D)** Message interval: 0.5 s, **(E)** Message interval: 1.0 s.

Figure 11 depicts the power consumption distribution for the action server node across 20 executions, varying the number of clients and message interval. At higher frequencies, it is visually evident that both the programming language and message frequency remain key determinants of power consumption. This trend is still noticeable at a 1.0-s message interval, although the difference between the two languages becomes less pronounced, varying by less than 0.1 W in executions with three clients. Unlike other communication server nodes, the C++ implementation appears to be more affected. However, this is inconclusive since it may be a visual misinterpretation, as the overall power consumption for each execution is lower compared to other communication patterns. The most plausible explanation is that, in other communication patterns, the client disconnects and reconnects to the server for every message exchange, whereas in *action* communication, only a new goal is sent, and feedback is received. It is important to note that this behavior is not an implementation issue but rather an inherent characteristic of this communication pattern.

##### 6.3.1.1 Statistical tests

**FIGURE 12:**
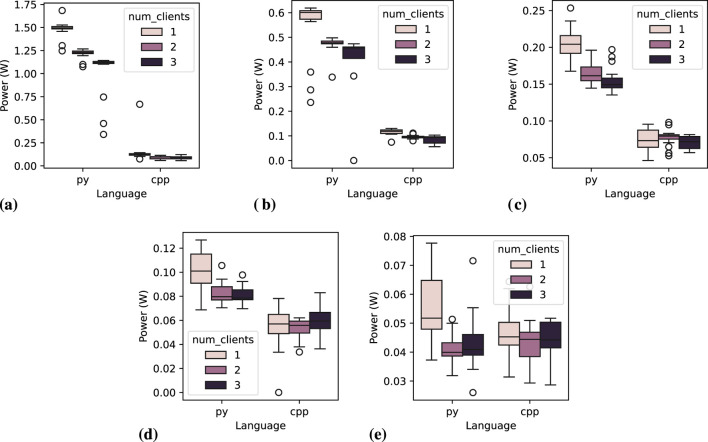
Power consumption distribution for one *action client* node across 20 executions, while varying the number of clients and message interval. **(A)** Message interval: 0.05 s, **(B)** Message interval: 0.1 s, **(C)** Message interval: 0.2 s, **(D)** Message interval: 0.5 s, **(E)** Message interval: 1.0 s.

The statistical tests indicate that, as for other communication patterns, both programming languages and message frequencies result in statistically significant differences in power consumption. The Kruskal–Wallis test results in $p=1.70\times 10^{-44}$ for Python and $p=1.35\times 10^{-54}$ for C++. However, varying the number of clients shows a slight statistical difference among Python groups, which is explained by Dunn’s test results, where groups 1 and 2 do not indicate a statistical difference, and the difference for group 3 is less expressive than for the communication patterns. In contrast, for C++, there is a statistically significant difference among all the groups, except at 0.2-s message interval. Further analysis, additionally grouping the data by message frequency reveals that all frequency measurements are statistically different among C++ groups, whereas none of the Python frequency measurements show statistical significance. This indicates that Python either is not holding the concurrent communication properly or it does it precisely well that measurements are not impacted with multi-client executions. The analysis of *action client* figures (Figure 12) gives details of the possible reasons for such behavior, which better aligns with the previous hypothesis.

#### 6.3.2 Action client

Table 11 presents the results of a single *action client* node operating at various frequencies and with different numbers of clients across 20 executions. A visual inspection reveals no observable influence of the number of clients on power consumption for C++. However, in Python, a consistent decrease in power consumption is observed as the number of clients increases, which differs from *action server*, and even the CPU usage consistently decreases with more clients. Another interesting and related observation is at 1.0-s message interval, when Python language seems to consume less energy than C++, except with runs with a single client. This consistent reduction in Python’s power consumption suggests that all measurements with one client require more power compared to those with two or three clients. This behavior is likely linked to multi-client architectural triggering, such as synchronization or multi-threading mechanisms, which are potentially activated in such scenarios.

**TABLE 11:** Comparative results of one *action client* node with different frequencies and number of clients over 20 executions.

Language	# Clients	Msg. Interval (s)	Avg. CPU utilization (%)	Avg. Memory utilization (KB)	Total CPU energy (J)	CPU power (W)
*C++*	1	0.05	0.59	2073	21.28	0.15
		0.1	0.45	2,117	21.97	0.12
		0.2	0.25	1984	14.22	0.07
		0.5	0.19	1946	10.52	0.05
		1.0	0.16	1864	9.01	0.05
	2	0.05	0.35	2,175	16.32	0.09
		0.1	0.36	2,192	17.72	0.1
		0.2	0.26	1983	14.53	0.08
		0.5	4.06	1932	9.00	0.05
		1.0	0.15	1965	8.34	0.04
	3	0.05	0.35	2,214	16.23	0.09
		0.1	0.33	2,152	15.83	0.09
		0.2	2.76	2073	12.60	0.07
		0.5	0.22	1975	11.37	0.06
		1.0	0.16	1837	8.56	0.04
*Python*	1	0.05	6.36	4,129	273.79	1.48
		0.1	2.08	3,817	94.33	0.55
		0.2	0.71	4,140	38.59	0.2
		0.5	0.34	3,519	19.3	0.1
		1.0	0.19	3,496	10.46	0.06
	2	0.05	5.59	4,140	225.14	1.22
		0.1	1.99	4,138	87.09	0.47
		0.2	0.58	4,142	31.23	0.17
		0.5	0.28	3,870	15.56	0.08
		1.0	0.15	3,693	7.96	0.04
	3	0.05	5.15	4,138	174.65	1.03
		0.1	1.59	4,138	63.47	0.36
		0.2	0.56	3,925	29.19	0.15
		0.5	0.29	3,688	15.77	0.08
		1.0	0.2	3,668	9.06	0.04

Figure 12 illustrates the distribution of mean power consumption for a single *action client* node over 20 executions, varying the number of clients and the message interval. The observations from Table 11 are corroborated by the sub-figures. Notably, disruptive measurements are evident for Python nodes when operating with two or three clients, as compared to a single client. Additionally, the power consumption gap between Python and C++ narrows as the message interval increases. At a 1.0-s message interval, Python measurements seem to be compatible with C++ ones.

In a practical context, where an action client might send navigation or manipulation tasks to a robot, having more than one client is generally unnecessary, except for the need for feedback messages by secondary nodes. Therefore, this configuration might be neglected, which could cause the unexpected behavior observed. Since the results focus on a single client (with monitoring limited to the last client), we re-executed the Python experiments to monitor all clients simultaneously. This approach aimed to verify whether any client exhibited anomalous behavior, which was not identified over a thorough log analysis.

##### 6.3.2.1 Statistical tests

The statistical tests confirm that programming language and message frequency are determinant factors, except for C++, where 0.05- and 0.1-s message intervals do not result in statistically significant differences in power measurements. For C++, different numbers of clients result in statistical differences on the measured power consumption, except between groups 2 and 3. For Python, the number of clients also leads to statistically different power measurements among all the groups. An additional Kruskal–Wallis between the two languages at 1.0-s interval indicates no statistical difference (p=0.92) between groups, confirming the visual assumption when analyzing Figure 12E, that both languages result in closely the same power consumption at low message frequency.

### 6.4 Comparison of communication pattern measurements

Since we experiment all the studied communication patterns with a controlled and homogeneous scenario, in this section, we compare their overall measurements, dividing them into *publisher/server* and *subscriber/client*.

#### 6.4.1 Publisher/server measurements

**FIGURE 13:**
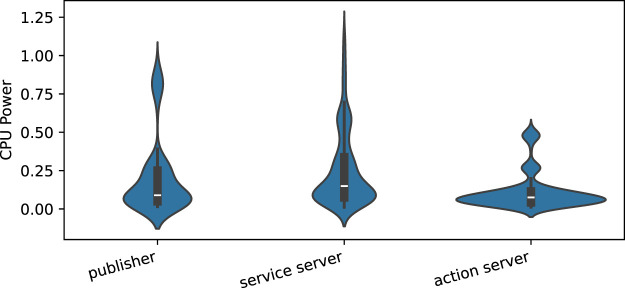
Power consumption distribution over all the combinations of independent variables for *publisher/server* nodes across the 20 executions.

Figure 13 illustrates the distribution of power consumption across all combinations of independent variables (configurations) for the *publisher/server* nodes over 20 executions. The plot reveals a consistent mean CPU power consumption across the different nodes, although there is an observable distinction across distribution variations. The *action server* presents the least variation in measurements, while the *service server* shows the greatest fluctuation.

##### 6.4.1.1 Statistical tests

The Shapiro-Wilk test of three groups (*publisher*, *service server*, and *action server*) indicates that their data are significantly non-normally distributed. Following this, the Kruskal–Wallis test was performed to compare the groups, which resulted in a highly significant result (*H* statistic = 227.22, $p=4.56\times 10^{-50}$ ), indicating substantial differences between the groups. Further analysis with Dunn’s *post hoc* test revealed that all pairwise group comparisons were statistically significant, with very small *p-values*. This confirms that despite the consistent mean values observed in Figure 13, the power consumption among the three nodes is significantly different.

#### 6.4.2 Subscriber/client measurements

**FIGURE 14:**
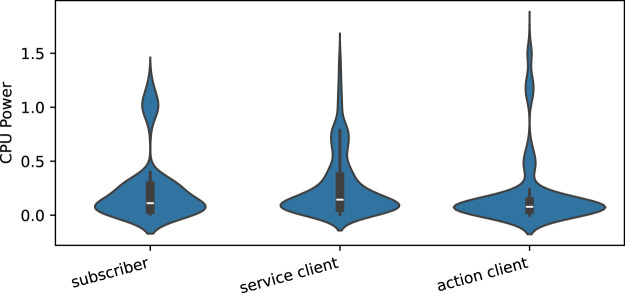
Power consumption distribution over all the combinations of independent variables for *subscriber/client* nodes across the 20 executions.

Figure 14 illustrates the distribution of power consumption across all combinations of independent variables (configurations) for *subscriber/client* nodes over the 20 executions. The graphs show a tighter distribution of values than those for *publisher/server* nodes, which also seem to result in closer mean values. Among the plots, the *service client* and *action client* show the most similar distributions, while the *subscriber* displays a slightly different pattern.

##### 6.4.2.1 Statisitcal tests

The Shapiro-Wilk test results indicate that the data for all three groups is likely not normally distributed. Therefore, the Kruskal–Wallis test was performed to assess differences among the groups. The test reveals a significant result (*H* statistic = 80.48, $p=3.34\times 10^{-18}$ ), indicating differences between the groups. Post-hoc analysis using Dunn’s test reveals significant pairwise differences among all the groups, with *action client* showing the most significant differences compared to the others. This confirms our observation about the *action client* distribution, although rejecting the hypothesis of power distributions being close, despite their consistent measurement means.

## 7 Discussion

In this section, we summarize and discuss further details of the experiment results, answer the research questions, and ponder about the impact of the findings of our investigation.

### 7.1 Summary of main findings

Here, we summarize the findings discussed as topics, which makes it easier for the reader to navigate through them.

• *C++ is the most efficient programming language*: C++ consistently outperformed Python in terms of energy efficiency and resource usage across all ROS two communication patterns. This was already expected since a previous study that bases this research (Pereira et al., 2021) has already revealed C and C++ superiority regarding energy efficiency. However, in the case of ROS, we expected a shrank difference since both language libraries (*rclpy* and *rclcpp*) share the same underlying programming and communication layers.
• *Message interval is a determinant factor*: Higher message frequencies led to increased power consumption in both C++ and Python, highlighting the importance of optimizing message intervals. The highest message rates experimented, i.e., 0.05-s and 0.1-s message intervals, recurrently led to overhead, indicating the it is important to limit the message exchange to higher rates, starting from four messages per second (0.2-s message interval).
• *The number of clients triggers unexpected behaviors*: Despite being less impactful, the number of clients is still a determining factor that must be considered in the design phase of ROS software systems. It also revealed potential architectural issues, such as for Python *action client* nodes, that result in unexpected low power measurements when scaling from one to two or three clients, which foster further investigation.
• *Python’s scalability is unpredictable*: Python exhibited less predictable and often unstable behavior as the number of clients increased, particularly at high frequencies. This suggests potential limitations in Python’s ability to handle demanding robotic communication scenarios efficiently.
• *Servers are directly impacted by different independent variables*: The number of clients significantly impacted power consumption on the server-side nodes, but the specific effects varied depending on the communication pattern.
• *Clients are less susceptible to the number of clients*: It is expected that the number of clients do not affect clients directly since it do not result in extra workload. However, some task from the underlying architecture or the server, possibly related to synchronization, seems to affect such nodes as well.
• *Experiments revealed potential issues*: The research suggests that the dependency of programming language (C++ or Python) on the underlying ROS two architecture (DDS middleware, client libraries) plays a crucial role in energy efficiency. For instance, unexpectedly, power consumption decreased as the number of clients increased on the client-side for services and actions. We could not identify any anomaly in the experiment executions after a careful investigation of logs and measurement data. A quick investigation on *rclpy* and *rclcpp* libraries does not reveal substantial evidence of such a behavior either.

### 7.2 Research question answers

The research questions are repeated here, avoid seeking fro them back in the document while reading their answers.

*RQ1: How is the energy efficiency of each ROS communication pattern?*

The statistical tests described in Section 6.4 demonstrate that the measurement data for the three communication patterns differ significantly, highlighting that each pattern has a distinct impact on energy efficiency. However, the mean values of the measurement distributions for the *publisher*/*server* and *subscriber*/*clients* patterns (Figures 13, 14, respectively) are closely aligned. Additionally, the violin plots in these figures reveal that most measurements, particularly for the *subscriber*/*clients*, are concentrated within a similar range. Given these findings, we recommend a careful design study when selecting a communication pattern. Nonetheless, we acknowledge that the patterns can likely be interchanged without significant consequences for energy efficiency, especially in the case of *publisher*/*subscriber* and *service* patterns.

*RQ2: How do the* *C++* *and* *Python* *implementations affect resource usage and energy consumption when handling different communication patterns among ROS nodes?*

The programming language is a determinant factor, where Python leads to higher power consumption through all the experiment results. This is an expected behavior based on recent research with programming languages for data structure algorithms (Pereira et al., 2021); however, the difference in power consumption among the two studied languages is surprising given the amount of underlying architectural layers shared by both language libraries. After the experiments, we strongly recommend the use of C++ for ROS two implementations, which can benefit scalability, reliability (due to a more predictable behavior), and energy/resource usage efficiency.

*RQ3: How does language efficiency scale over different frequencies of communication and number of clients?*

We observe that message frequency is a strong determinant factor of energy efficiency that should be carefully considered when designing ROS systems. High message frequencies demonstrate to lead to resource overhead, triggering unstable behaviors, particularly in *Python* nodes when more than one client connects with the servers, which significantly compromises scalability. While the number of clients is a less critical factor compared to the programming language and message interval, it remains an important consideration. It impacts not only energy efficiency but also introduces unexpected behaviors, such as those observed with *actions*. These behaviors may indicate poor design choices, especially since multi-client setups are often impractical in most scenarios where such patterns are applied. Therefore, we recommend a careful design when considering a multi-client ROS system.

### 7.3 Impact of the findings

The findings of this research have significant implications for the development and operation of real-world robotic systems using ROS. In the sequence, we discuss some of the key implications. As an excerpt of real-world robotic projects, we analyzed a list of 946 carefully curated ROS two repositories on GitHub^21^, obtained from a separate ongoing research project by the authors. Those projects were selected considering quantitative criteria that make the projects to be representative (such as number of forks, number of followers and contributors, and size), which numbers are included in the dataset.

Due to its high level of abstraction, Python is a particularly attractive language for newcomers to programming, which can also be the case of those starting with robotics and ROS. However, as these results suggest, the widespread use of Python can lead to significant energy inefficiencies, impacting projects that rely on battery-powered robots, besides the environmental side-effects. Among the real-world repositories in the referenced dataset, 291 (30%) utilize Python as their primary language. This represents a substantial number of projects, which can be directly used, reused as packages, or serve as models for future implementations. Disseminating this paper’s findings to both, academic and industry communities, can lead to more informed decisions regarding programming language selection in robotics projects, which will potentially benefit resource and energy efficiency.

The direct correlation between message frequency and power consumption highlights the critical need for optimization of message intervals within ROS two systems. Robotics developers should attempt to minimize message frequencies while ensuring that essential system functionality remains unimpaired. To understand this impact, we manually queried the first repositories in the real-world dataset. A superficial analysis reveals that service calls tend to be less affected by high frequencies since they are usually triggered by events, such as in the *main_camera_node.cpp* file of *cyberdog_ros2* project^22^, which triggers camera-related services on *configure* event and resets it on *clean up* event. However, for *publisher*/*subscriber* cases, the impact tends to be more critical. A critical example of this is the *webots_ros2* project^23^, where the *epuck_node.py* file implements a node with multiple subscriptions to different topics, and do not pace the communication with any delay or sleep. In such cases, message frequency is primarily dictated by factors such as the execution time of the whole algorithm. For simple algorithms, these execution times can result in fractions of seconds, leading to excessively high message frequencies, a significant concern in our experiment results, particularly for Python-based projects.

The final factor, and the least impactful, is the number of clients. This scenario is more commonly associated with the *publisher*/*subscriber* communication model, given its sensor-based nature. In a manual analysis of the first 20 projects in the dataset, we identified only one project, *ros2_canopen* ^24^, with more than one subscriber. In this project, the *node_name + rpdo* topic is subscribed to by two different test nodes (*test_node* and *simple_rpdo_tpdo_tester*), while the *low_level/joint_states* topic is subscribed to by the *noarm_squat* and *wiggle_arm* nodes. Despite multiple clients seeming to be less common, the repositories’ manual inspection supports the legitimacy of our concerns regarding multiple clients and suggests that our observations can also contribute to thoughtful designs that take the number of clients into account.

### 7.4 Open issues

Unfortunately, the results reveal a few issues whose sources we were unable to identify. We outline these issues below to encourage further investigations, as their resolution and deep investigation lie outside the scope of this paper. We confirm that they are not related to issues in our algorithms or their executions, which have already been discussed in the previous sections.

1. The Python *publisher* and *subscriber* mechanisms exhibit high variability at elevated frequencies, suggesting some form of overhead that leads to unpredictable power consumption.
2. The C++ *service server* demonstrates an initial power increase when the number of clients rises from one to two. However, the power consumption stabilizes as the number of clients increases from two to three. This behavior, distinct from that observed in Python, suggests a one-time synchronization method for handling multiple clients.
3. While the Python *service server* shows an increase in power consumption as the number of clients grows, the Python *service clients* exhibit a consistent decrease in power consumption. This may indicate challenges faced by the server in addressing all client requests, although this hypothesis is not supported by manual log analysis.
4. At low message frequencies, the *action* pattern results in similar power consumption for both languages. This consistency is not observed for other communication patterns.

## 8 Threats to validity

In this section, we discuss potential threats to the validity of our experiments, outline considerations, and describe how we address each of them.

### 8.1 External validity

One limitation lies in the simplicity of the messages exchanged between ROS two nodes in our experiments. We focused on plain text messages in the *publisher*/*subscriber* pattern, which are less complex compared to sensor messages like PointCloud ^25^ or geometry-based messages^26^. However, our results show consistent behavior across various configurations and communication patterns, with significant differences between configurations, suggesting that the experimental variables likely impact systems using more complex message types. Additionally, prior work (Albonico et al., 2024) involving more diverse message types indicates that at least the number of clients plays a critical role, reinforcing the applicability of our findings. To facilitate further exploration, our replication package supports extensions to other communication patterns and message types.

To mitigate potential biases due to the representativity of the implemented ROS two nodes, we based our implementation on official ROS two tutorials. This ensures relevance and applicability to a wide range of general-purpose applications since they may work as a template for different types of applications worldwide. Moreover, our study uses ROS 2 Humble, an active distribution supported until 2027, enhancing the relevance and timeliness of our findings.

### 8.2 Internal validity

We ensured internal validity by maintaining a strictly controlled experimental environment, minimizing the influence of variations in system load, background processes, or hardware inconsistencies. All experiments were conducted using the same tools and environment and repeated twenty times to account for variability. For CPU and memory usage measurements, we relied on widely used Python libraries. Energy consumption measurements were conducted using a tool extensively validated in prior studies (Kamatar et al., 2024; Nahrstedt et al., 2024; Makris et al., 2024). This rigorous approach minimizes confounding factors and strengthens the reliability of our conclusions.

The use of Docker in our experimental setup introduces a potential internal threat, as it may slightly influence energy measurements due to its resource isolation and runtime overhead. These effects could introduce systematic measurement biases, affecting the accuracy and reproducibility of our results. This is mitigated by a controlled execution, where we compare the energy usage of different ROS two communication methodologies within a comparable environment. This approach helps mitigate the potential impact of Docker-induced variations, as any overhead introduced by containerization would be present across all experimental conditions. Since we are primarily interested in how different communication patterns compare to each other in terms of energy consumption, rather than the exact power drawn by each, minor variations introduced by Docker do not compromise the validity of our conclusions.

### 8.3 Construct validity

Our experiments were designed with well-established metrics that align with the goals of this research (Pereira et al., 2017; Hähnel et al., 2012). Power consumption, a primary metric, directly measures the rate of energy usage while running ROS nodes, capturing nuanced differences in energy efficiency attributable to language-specific and architectural factors. This metric is independent of confounding variables such as execution time, ensuring that observed effects are solely related to energy efficiency. Furthermore, all findings were validated using a robust statistical testing strategy, ensuring the reliability of our conclusions and alignment with the study’s objectives.

## 9 Related work

The energy efficiency of software has received significant attention in recent years, particularly concerning the deployment infrastructures and programming languages utilized in various applications. This aligns closely with the objectives of our research, which aims to investigate the energy efficiency of programming languages within the Robot Operating System (ROS) ecosystem. Notably, a comprehensive search across major research databases revealed a gap in the literature, as no previous studies have specifically addressed the energy efficiency of programming languages in the context of ROS.

The work by Pereira et al. (2017) stands out as the most related to our investigation. Their extensive study evaluated energy consumption across a variety of algorithms implemented in different programming languages, producing a ranking based on energy efficiency. While their findings provide valuable insights, the algorithms they examined were executed natively, without the influence of middleware or frameworks, contrasting with our focus on ROS-specific algorithms that operate within an active ROS stack. This distinction is crucial, as the architecture and operational context of ROS can significantly impact energy consumption metrics.

Other studies have explored the energy efficiency of programming languages in different contexts. For instance, Kholmatova (2020) examined the impact of programming languages on energy consumption for mobile devices, highlighting how language choice can influence energy efficiency. Similarly, Abdulsalam et al. (2014) investigated the effects of language, compiler optimizations, and implementation choices on program energy efficiency, emphasizing the importance of these factors in software development. Furthermore, research by Holm et al. (2020) focused on GPU computing with Python, analyzing performance and energy efficiency, which underscores the relevance of programming paradigms in energy consumption.

In our previous work (Albonico et al., 2024), we investigated the energy efficiency of ROS nodes implemented in C++ and Python. Their study focused on the publisher-subscriber communication pattern and assessed the power consumption of ROS nodes in both languages. The results demonstrated that C++ nodes exhibit superior energy efficiency compared to Python nodes, particularly in scenarios with multiple subscribers. This difference was attributed to the architecture of the client libraries and the native multi-threading capabilities of C++. However, compared to this paper, their study considered fewer independent variables, followed a less systematic methodology, and provided only preliminary results with limited discussion.

## 10 Conclusion

Our study provides a comprehensive analysis of the energy efficiency of ROS two communication patterns, revealing that C++ implementations consistently outperform Python in terms of energy efficiency and resource usage. Message frequency significantly influences power consumption, while the number of clients has a less predictable impact, particularly for Python. These findings have significant implications for real-world robotic systems, guiding programming language choices, message frequency optimization, and system architecture considerations. Therefore, our research contributes to a better understanding of energy efficiency in ROS 2, promoting the development of greener robotic software.

Despite the consistency of our findings across various configurations and communication patterns, there are still a few open issues that can be further investigated. For example, further research can explore the impact of message type complexity, particularly with other types of messages. We also plan to examine the specific synchronization or multi-threading mechanisms in both C++ and Python service servers and clients to understand the observed power consumption trends as the number of clients increases. Finally, another possible extension of this research, is to analyze the action pattern to determine why it results in similar power consumption for both C++ and Python at low message frequencies.

## Data Availability

The datasets presented in this study can be found in online repositories. The names of the repository/repositories and accession number(s) can be found in the article/supplementary material.

## Footnotes

1
https://wiki.ros.org/ROS/Patterns/Communication

2
https://docs.ros.org/en/galactic/Tutorials.html

3
https://greencompute.uk/Measurement/RAPL

4
https://answers.ros.org/question/295426/why-is-pubsub-the-ideal-communication-pattern-for-ros-or-robots-in-general-instead-of-requestresponse/

5
https://github.com/ros2/rclcpp

6
https://github.com/ros2/rclpy

7
https://docs.ros.org/en/foxy/Concepts/About-Executors.html

8
https://www.intel.com/content/www/us/en/developer/articles/technical/software-security-guidance/advisory-guidance/running-average-power-limit-energy-reporting.html

9
https://realpython.com/python-gil/

10
http://wiki.ros.org/ROS/Tutorials

11
https://chatgpt.com/

12
https://index.ros.org/p/joy/

13
https://github.com/IntelAgir-Research-Group/frontiers-robotics-ai-rep-pkg/blob/main/data-analysis/repos.csv

14
https://github.com/IntelAgir-Research-Group/frontiers-robotics-ai-rep-pkg

15
https://docs.ros.org/en/humble/index.html

16
http://wiki.ros.org/roscpp/Overview/Callbacks%20and%20Spinning

17
https://www.intel.com/content/www/us/en/developer/articles/technical/software-security-guidance/advisory-guidance/running-average-power-limit-energy-reporting.html

18
https://pypi.org/project/psutil/

19
https://github.com/IntelAgir-Research-Group/frontiers-robotics-ai-rep-pkg/tree/main/

20
https://github.com/ros2/rmw/issues/372

21
https://github.com/IntelAgir-Research-Group/frontiers-robotics-ai-rep-pkg/blob/main/data-analysis/repos.csv

22
https://github.com/MiRoboticsLab/cyberdog_ros2/

23
https://github.com/cyberbotics/webots_ros2

24
https://github.com/ros-industrial/ros2_canopen

25
https://docs.ros.org/en/noetic/api/sensor_msgs/html/msg/PointCloud.html

26
https://docs.ros2.org/foxy/api/geometry_msgs/index-msg.html
